# RPE-specific MCT2 expression promotes cone survival in models of retinitis pigmentosa

**DOI:** 10.1073/pnas.2421978122

**Published:** 2025-04-03

**Authors:** Laurel C. Chandler, Apolonia Gardner, Constance L. Cepko

**Affiliations:** ^a^Department of Genetics, Blavatnik Institute, Harvard Medical School, Boston, MA 02115; ^b^Department of Ophthalmology, Harvard Medical School, Boston, MA 02115; ^c^HHMI, Chevy Chase, MD 20815; ^d^Virology Program, Harvard Medical School, Boston, MA 02115

**Keywords:** retinitis pigmentosa, gene therapy, retinal pigment epithelium, metabolism

## Abstract

Retinitis pigmentosa (RP) is the most common cause of inherited retinal degeneration worldwide. It is characterized by the sequential death of rod and cone photoreceptors, the cells responsible for night and daylight vision, respectively. Although the expression of most RP genes occurs only in rods, there is a secondary degeneration of cones. One possible mechanism of cone death is metabolic dysregulation. Photoreceptors are highly metabolically active, consuming large quantities of glucose and producing substantial amounts of lactate. The retinal pigment epithelium (RPE) mediates the transport of glucose from the blood to photoreceptors and, in turn, removes lactate, which can influence the rate of consumption of glucose by the RPE. One model for metabolic dysregulation in RP suggests that following the death of rods, lactate levels are substantially diminished causing the RPE to withhold glucose, resulting in nutrient deprivation for cones. Here, we present adeno-associated viral vector-mediated delivery of monocarboxylate transporter 2 (MCT2, *Slc16a7*) into the eye, with expression limited to RPE cells, with the aim of promoting lactate uptake from the blood and encouraging the passage of glucose to cones. We demonstrate prolonged survival and function of cones in rat and mouse RP models, revealing a possible gene-agnostic therapy for preserving vision in RP. We also present the use of fluorescence lifetime imaging-based biosensors for lactate and glucose within the eye. Using this technology, we show changes to lactate and glucose levels within MCT2-expressing RPE, suggesting that cone survival is impacted by changes in RPE metabolism.

## Significance

The leading cause of inherited retinal degeneration is retinitis pigmentosa. Due to the large number of disease-causing genes, developing a specific gene therapy for each is challenging. An alternative strategy is to target pathways common across disease gene families. Loss of daylight vision due to cone photoreceptor degeneration is one common pathway. Cone degeneration is proposed to be due, in part, to loss of nutrient delivery from the retinal pigment epithelium (RPE). Here, we develop a gene therapy expressing monocarboxylate transporter 2 specifically in the RPE and show that it prolongs cone survival and function. We also demonstrate that fluorescent lifetime imaging-based biosensors can help track the metabolism of ocular cell types, potentially informing the design of future therapeutics.

Retinitis pigmentosa (RP) is a group of monogenic, heritable neurodegenerative diseases that result in loss of vision for 1 in 4,000 people worldwide (1). There are a significant number of genes (~100) associated with RP (RetNet). RP genes are primarily expressed within rods, resulting in their degeneration and the loss of night vision. As the disease progresses, there is a secondary loss of cones, resulting in deterioration of daylight color vision. Although the precise mechanisms remain unclear, various studies have suggested that inflammation (2–4), oxidative damage (5–8), and metabolic dysregulation (9–13) each contribute to the degeneration of cones. A gene-agnostic treatment strategy that addresses potentially common mechanisms of cone degeneration would benefit a significantly larger patient population than addressing each disease gene individually.

Rod and cone photoreceptors are highly metabolically active and rapidly metabolize glucose via aerobic glycolysis to generate substantial quantities of lactate (14–17). As the photoreceptor layer is not vascularized, photoreceptors rely on the retinal pigment epithelium (RPE) to mediate the passage of glucose from the choroidal vasculature to the retina (18). Lactate is imported back across the apical membrane of the RPE through monocarboxylate transporter 1 (MCT1, *SLC16A7*) and into the blood through the basolateral membrane transporter MCT3 (*SLC16A8*) (19). A portion of this lactate is retained by the RPE and is converted to pyruvate by lactate dehydrogenase B (LDHB), thus fueling its own metabolic needs (17). This reaction has been shown to suppress glycolysis in the RPE by depleting the available NAD^+^, thus allowing for a greater pool of glucose to be available for transport to photoreceptors (17).

A model for metabolic dysregulation in RP suggests that the loss of rods substantially reduces the level of lactate available to the RPE. As the suppressive action of lactate would be lost, the RPE may begin withholding glucose for its own glycolysis, thus depriving the surviving cones of nutrients (9, 12). We aimed to encourage the RPE to take up lactate from the blood and relinquish glucose to the surviving cones, by expressing the high-affinity lactate transporter, MCT2, specifically within RPE cells using an adeno-associated viral (AAV) vector. We hypothesized that the RPE would be unable to import sufficient lactate from the choroid with its basolateral transporter, MCT3, as its affinity for lactate (*K*_m_ ≈ 6 mM) is too low relative to blood lactate concentrations (2.5 to 4.6 mM) for efficient uptake from the blood (20–22). The affinity of MCT2 is, however, much higher (*K*_m_ ≈ 0.7 mM) and theoretically should be able to allow the RPE to take up a greater amount of lactate in these conditions (23).

Here, we demonstrate that AAV vector-mediated delivery of MCT2 into both rat and mouse models of RP, with expression restricted to the RPE, increased cone cell survival and function across four different disease-causing mutations. We also present the use of fluorescence lifetime imaging microscopy (FLIM)-based biosensors in the eye to measure the localization, uptake, and metabolism of lactate and glucose (24, 25). We found significantly greater intracellular lactate levels and a greater accumulation of glucose in RPE cells overexpressing MCT2. Together, these data suggest that increasing lactate uptake into the RPE can promote cone survival, supporting the model of insufficient or dysregulated nutrient distribution in RP as a factor in cone degeneration, and encouraging the development of therapies to address this shortcoming.

## Results

### RPE-Specific MCT2 Expression Leads to Increased Cone Survival in an RP Rat Model.

**Fig. 1.**
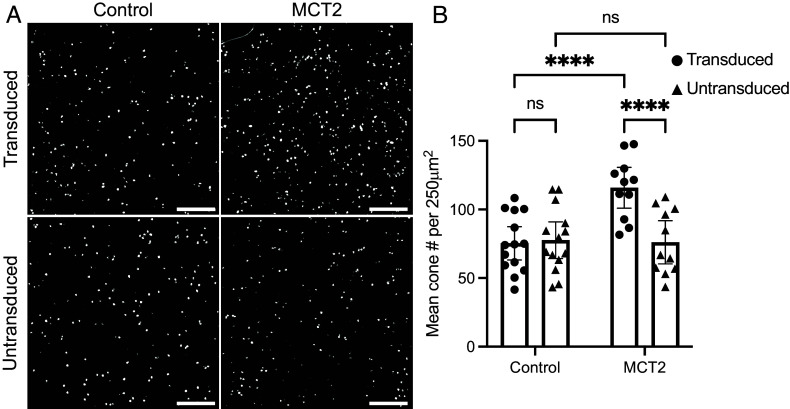
RPE-specific monocarboxylate transporter 2 (MCT2) expression increases cone survival in a RP rat model. S334ter line-3 rats were subretinally injected with red fluorescent beads alone (control) or coinjected with AAV8.Best1.MCT2 (MCT2). Cones were identified using in situ hybridization against arrestin-3 (*Arr3*) at P183-191. (*A*) Representative 500 μm^2^ images taken from the mid-periphery of transduced and untransduced regions from control and MCT2 retinae. (Scale bar, 100 μm.) (*B*) Absolute cone counts averaged across four 250 μm^2^ regions within the transduced and untransduced regions of control and MCT2 samples (±95% CI; control, n = 14; MCT2, n = 11). ****P ≤ 0.0001 (two-way repeated measures ANOVA with Šídák’s multiple comparison test). The density of cones in a wild-type Sprague-Dawley rat at approximately P84 is 407.5 ± 47.07 per 250 μm^2^ (n = 8; average of both eyes).

To encourage glucose release and cone survival, we aimed to promote RPE lactate uptake by expressing the high-affinity lactate transporter, MCT2. We generated an AAV serotype 8 vector expressing MCT2 (*Slc16a7*) under the control of the RPE-specific promoter bestrophin-1 (Best1) (AAV8.Best1.MCT2) (26). To assess the effect of RPE-specific MCT2 expression on cone survival, we initially performed paired subretinal injections in the RP rat model, S334ter line-3, which expresses a termination codon at residue S334 in the rhodopsin gene on a Sprague-Dawley background (27). Neonatal rat pups were subretinally injected with AAV8.Best1.MCT2 together with red fluorescent beads, which were used to approximately mark the transduced region; paired control eyes were injected with beads only. At postnatal days 183 to 191 (P183-191), retinae were harvested, and surviving cones were stained using hybridization chain reaction RNA fluorescence in situ hybridization (HCR RNA-FISH) for retinal cone arrestin-3 (*Arr3*). By this timepoint, the rod photoreceptors would have degenerated and the outer nuclear layer reduced to less than one complete row in the most degenerated regions (27); we therefore observed no residual pattern of cone survival across the retina. For this reason, we quantified cone numbers within each retina in bead positive and negative regions (*SI Appendix*, Fig. S1). In eyes coinjected with AAV8.Best1.MCT2, there was a statistically significant increase in the number of surviving cones in transduced relative to untransduced areas in the same eye (two-way repeated measures ANOVA, *P* < 0.0001) (Fig. 1). There was also a significant increase between the transduced regions of control and AAV8.Best1.MCT2-injected eyes (*P* < 0.0001). Although AAV8.Best1.MCT2 significantly increased the number of cones, the numbers were still significantly less than in a wild-type Sprague-Dawley rat. There was no significant difference between the transduced and untransduced regions of control eyes (*P* = 0.8873), nor between the untransduced regions of the control and AAV8.Best1.MCT2-injected eyes (*P* = 0.9832). Overall, the data demonstrate that RPE-specific MCT2 expression can preserve cone survival in a rat model of RP.

### RPE-Specific MCT2 Expression Leads to Increased Cone Survival and Function in RP Mouse Models.

**Fig. 2.**
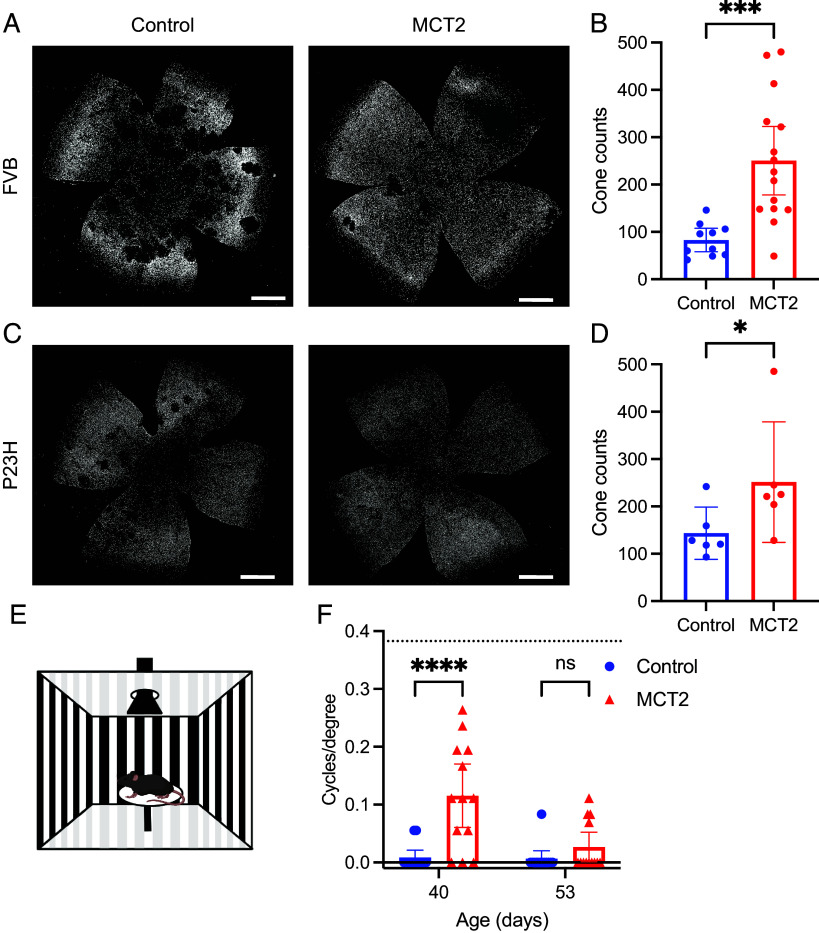
RPE-specific MCT2 expression increases cone survival and function in RP mouse models. (*A* and *B*) FVB mice were subretinally injected with AAV8.RedO.H2BGFP alone (control) or coinjected with AAV8.Best1.MCT2 (MCT2). (*A*) Representative image of P70 retinal flatmount with GFP^+^ cones from control or MCT2-injected eyes. (Scale bar, 750 μm.) (*B*) Quantification of the number of GFP^+^ cones in the central retina (±95% CI; control, n = 10; MCT2, n = 15). ***P ≤ 0.001 (unpaired *t* test). (*C* and *D*) P23H mice were subretinally injected with control or MCT2. (*C*) Representative image of P80 retinal flatmount with GFP^+^ cones from control or MCT2-injected eyes. (*D*) Quantification of the number of GFP^+^ cones in the central retina (±95% CI; n = 6). *P ≤ 0.05 (Mann–Whitney test). The density of cones in a wild-type C57BL/6 mouse at approximately P76 is 8,978 ± 1,405 (n = 3). (*E*) Schematic of the optomotor setup. (*F*) Visual acuity of paired control and MCT2 P23H eyes at P40 and P53 as measured by the number of cycles/degree using the optomotor assay (±95% CI; n = 13). ****P ≤ 0.0001 (two-way ANOVA with Šídák’s multiple comparison test). The dotted line represents the mean visual acuity of C57BL/6 mice at P56 (n = 9).

We also aimed to assess the effect of RPE-specific MCT2 expression on cones in RP mouse models. Subretinal injections were performed in neonatal FVB mice, an albino mouse strain homozygous for the retinal degeneration 1 allele of *Pde6b^rd1^*. As the red fluorescent beads used in our rat models seemed to underestimate the transduced area, we used an AAV8 vector expressing GFP fused to histone H2B under the control of a cone-specific human red opsin (RedO) promoter to label surviving cone nuclei (AAV8.RedO.H2BGFP). Although this vector should express specifically in cones, we see expression of H2BGFP in RPE when codelivered with a vector that expresses in the RPE, here AAV8.Best1.MCT2 (*SI Appendix*, Fig. S2). This is likely due to the concatenation or recombination of the two vectors in the RPE. This observation demonstrates the delivery of both vectors in a given area of the RPE and retina. Neonatal FVB mice were contralaterally injected with either AAV8.RedO.H2BGFP alone, or coinjected with AAV8.Best1.MCT2. Unlike the S334ter rat model, retinal degeneration in mice typically begins in the central retina at the optic nerve head and progresses toward the periphery; we therefore restricted our quantification to the center. When comparing GFP^+^ cone nuclei in AAV8.Best1.MCT2-injected and control retinal flatmounts at P70, there was a notable increase in the number of surviving cones in the central retina of AAV8.Best1.MCT2-injected mice (Fig. 2*A*). Furthermore, in eyes injected with AAV8.Best1.MCT2, there was a notable absence of what appears to be “craters” or lack of cones in scattered, circular areas, a prominent feature in this FVB strain (28). Upon quantification of the number of GFP^+^ cones in the central retina, there was a significant increase in AAV8.Best1.MCT2-injected eyes compared to control (unpaired *t* test, *P* = 0.0007) (Fig. 2*B*).

The model for metabolic dysregulation suggests a common mechanism of secondary cone loss across many disease-causing mutations of RP. We therefore assessed the effect of MCT2 expression in the P23H strain, a pigmented RP mouse model with a P23H mutation in the rhodopsin gene on a C57BL/6 background. We subretinally injected neonatal P23H mice with either AAV8.RedO.H2BGFP alone, or coinjected with AAV8.Best1.MCT2. Retinae were harvested at P80 and demonstrated an increase in the number of central cones (Fig. 2*C*). Furthermore, there appeared to be a small number of craters in control eyes, which were absent in AAV8.Best1.MCT2-injected retinae. Upon quantification, there was a statistically significant increase in the number of cones compared to control (Mann–Whitney test, *P* = 0.0455) (Fig. 2*D*). Similarly to the rats, although AAV8.Best1.MCT2 increased the number of cones, the numbers were still significantly less than in a wild-type C57BL/6 mouse. Overall, this suggests RPE-specific MCT2 expression improves cone survival in rats and mice, with three different RP-causing mutations.

The data demonstrate that MCT2 expression in the RPE of RP mouse models can increase survival of cone photoreceptors. However, to effectively provide vision, cone function must be preserved. Cone function was assessed in RP mouse models in paired eyes injected with and without AAV8.Best1.MCT2 by measuring the optomotor reflex, which functions as a readout for visual acuity (Fig. 2*E*). Several studies have suggested that the optomotor reflex in albino mice is absent or disrupted, therefore we were unable to assess visual acuity in FVB mice (29–31); we were also unable to assess optomotor reflex in rats due to housing constraints. We were able to assess a response in the pigmented P23H strain and, at P40, there was a significant retention of cone function in AAV8.Best1.MCT2-injected eyes compared to paired control eyes (two-way ANOVA, *P* < 0.0001), although it was not at the level of a healthy C57BL/6 mouse (Fig. 2*F*). A large amount of variability was observed in the optomotor response of the AAV8.Best1.MCT2-injected mice, likely due to at least two factors. One is that the subretinal injections are inherently variable, i.e., some animals may have a greater level of transduction than others and may therefore have increased visual acuity. The second is that behavioral assays show animal-to-animal variability. As the assay was performed when almost all the control eyes had lost their cone function, the same level of spread in the data was not seen in these samples. Despite MCT2 expression preserving cone function, visual acuity continued to diminish and by P53, there was no longer a significant difference (*P* = 0.4464). These data demonstrate that RPE-specific MCT2 expression in a RP mouse model significantly increases the duration of cone-mediated vision.

### Localization of MCT2 Protein in the RPE.

**Fig. 3.**
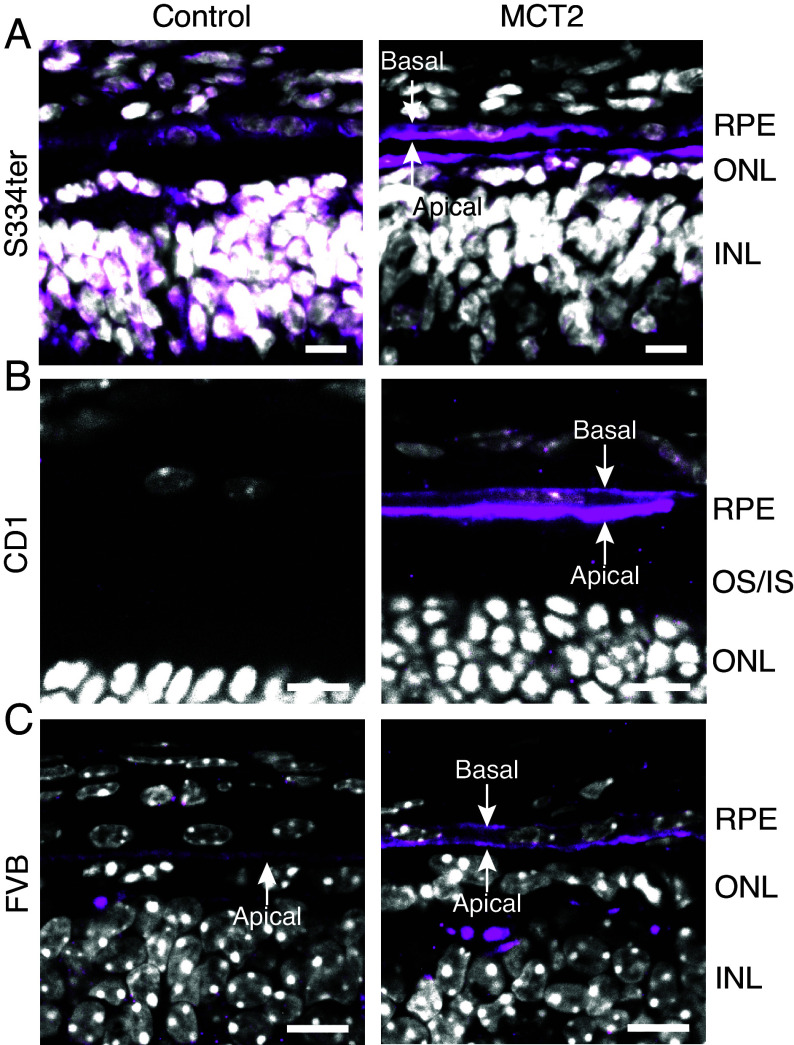
MCT2 expression on RPE cell membranes following subretinal injections with AAV8.Best1.MCT2. Representative section from a (*A*) P24 S334ter rat, (*B*) P42 FVB mouse, and (*C*) P46 CD1 mouse subretinally injected with (MCT2) and without (control) AAV8.Best1.MCT2. All sections stained with MCT2 antibody (magenta) and DAPI (white). (Scale bar, 10 μm.) Sections show MCT2 localization to the basal and apical membrane of RPE cells. OS/IS (outer segment/inner segment), ONL (outer nuclear layer), and IPL (inner plexiform layer).

The ancillary proteins basigin and embigin have been shown to play an important role in trafficking of MCTs to the plasma membrane. Basigin has been shown to preferentially act as a chaperone for both MCT1, which is localized to the apical membrane of RPE cells, and MCT3, which is localized to the basolateral membrane (32, 33). However, the mechanism leading to their localization to opposing RPE membranes remains unclear. The ancillary protein for MCT2 has been suggested to be embigin (34, 35). However, a recent study suggested that MCT2 can be transported to the plasma membrane without a chaperone (36). To determine whether MCT2 was expressed on the RPE plasma membrane following AAV8.Best1.MCT2 injections, we analyzed localization in S334ter rats (Fig. 3*A*), wild-type CD1 mice (Fig. 3*B*), and degenerate FVB mice (Fig. 3*C*) using immunohistochemistry. MCT2 expression was observed on both the apical and basal membranes of the RPE cell in all AAV8.Best1.MCT2-injected samples, demonstrating that it was transported to both cell surfaces. MCT2 was also observed in the outer plexiform layer of both control and AAV8.Best1.MCT2-injected samples, which is consistent with its expression in cone bipolar cells, as suggested by single-cell RNA sequencing (37).

### Effect of RPE-Specific MCT2 Expression on the Health of Retinal Tissue.

Previously, our group found that AAV vectors which express within the mouse RPE can induce toxic effects (38). We therefore aimed to determine whether the AAV8.Best1.MCT2 vector induced similar changes. RPE flatmounts were stained with phalloidin, which labels F-actin filaments just beneath the cell membrane and reveals the hexagonal shape of healthy RPE cells. In wild-type Sprague-Dawley rats infected with AAV8.Best1.MCT2, only minor changes to RPE morphology were observed (*SI Appendix*, Fig. S3), suggesting that RPE-specific expression of MCT2 did not induce significant toxic effects.

Although the AAV8.Best1.MCT2 vector did not induce significant toxicity in rats, there did appear to be some toxic effects in mice. At P40, FVB mouse eyes subretinally injected with AAV8.Best1.MCT2 were harvested for staining of the RPE with phalloidin. In control eyes, the RPE showed signs of disruption due to retinal degeneration (*SI Appendix*, Fig. S4). However, in AAV8.Best1.MCT2-injected eyes, there were additional morphological changes in the RPE, consistent with AAV-induced toxicity, including the enlargement of cells and the presence of stress fibers (*SI Appendix*, Fig. S4) (38). This observation indicates that mice were more sensitive to RPE-specific AAV.Best1.MCT2 expression than rats, despite receiving a lower dose than rats. Nonetheless, there were significant improvements in cone survival and function in mice.

### Metabolic Changes in the MCT2-Expressing RPE.

The rationale behind delivering MCT2 into RPE cells was to increase lactate uptake and prevent retention of glucose. To probe this mechanism, we utilized FLIM-based biosensors that specifically detect lactate and glucose. Fluorescence lifetime is the average time between photon excitation and emission, and, when used together with two-photon technologies, can robustly quantify the levels of a given molecule within a tissue. The benefit of lifetime measurements compared to fluorescence intensity readouts is that these measurements are independent of biosensor expression level and have reduced pH sensitivity (39). We generated AAV vectors expressing the FLIM-based lactate sensor, LiLac, and the glucose sensor, GlucoSnFR-TS, under the control of the RPE-specific Best1 promoter (24, 25). The LiLac sensor is composed of the lactate binding protein TlpC from *Helicobacter pylori*, which is inserted within the mTurquoise2 fluorophore sequence with the N and C termini of TlpC connected by flexible linkers (24). TlpC can generate a large conformational change upon binding to lactate which in turn causes lifetime shifts in the mTurquoise2 fluorescent protein. The bound version of LiLac has a shorter lifetime value compared to the unbound. In contrast, GlucoSnFR-TS is a T-sapphire fluorescent protein inserted between residues 326 and 327 of *Thermus thermophilus* glucose binding protein and has a longer lifetime value when bound (25).

**Fig. 4.**
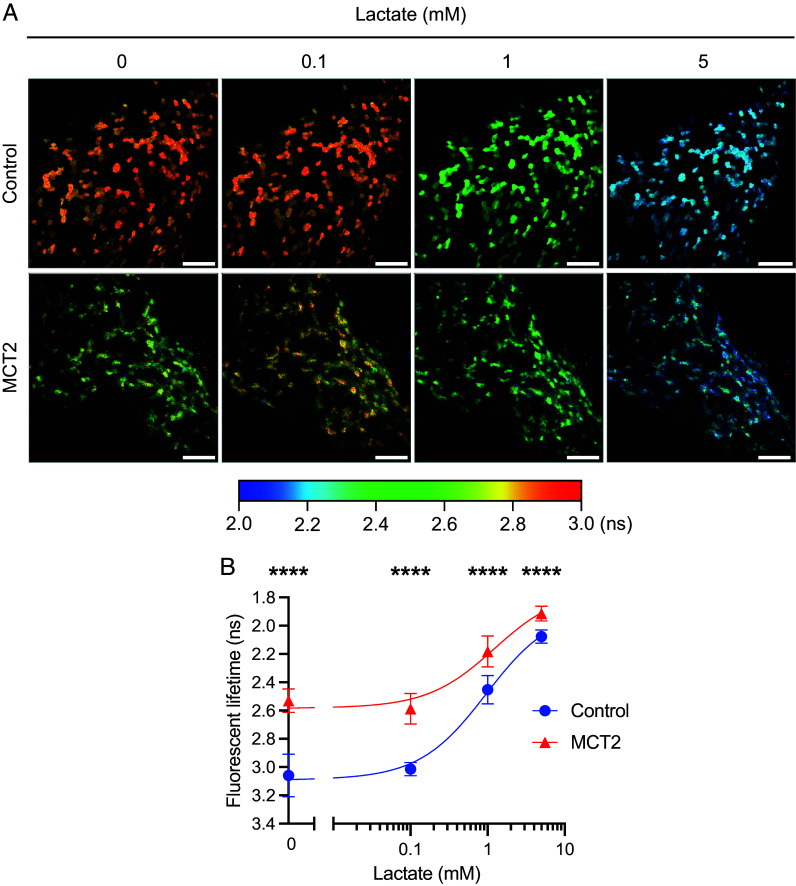
MCT2 overexpression leads to increased intracellular lactate in the RPE. Neonatal FVB mice were subretinally injected with AAV8.Best1.LiLac alone (control) or coinjected with AAV8.Best1.MCT2 (MCT2). At P20-21, eyecups (RPE, choroid, and sclera) were harvested and RPE cells imaged en face using two-photon FLIM. (*A*) Representative lifetime images from control and MCT2 samples sequentially incubated with 0, 0.1, 1, or 5 mM lactate. Color scale represents increasing lifetime in nanoseconds (ns), which corresponds to decreased intracellular lactate concentrations. (Scale bar, 100 μm.) (*B*) Lifetime at each lactate concentration (±SD; control, N_mice_ = 4; N_regions_ = 12; MCT2, N_mice_ = 6, N_regions_ = 16). Y-axis reversed to reflect rise in lactate concentration with decreasing lifetime and data fit with a dose–response curve. ****P ≤ 0.0001 (two-way ANOVA with Šídák’s multiple comparison test).

Initially, to assess the levels of lactate within MCT2-expressing RPE, we performed subretinal injections of AAV8.Best1.LiLac with and without AAV8.Best1.MCT2 into neonatal FVB mice. Three weeks later, the eyes were harvested and dissected, the retinas were removed, and the eyecups containing the RPE and choroid were flattened and immediately imaged using a two-photon fluorescent lifetime microscope to visualize lifetime changes in the RPE. The RPE tissue was incubated in Kreb’s ringer bicarbonate (KRB) buffer without glucose but with increasing concentrations of lactate (0, 0.1, 1, and 5 mM) that reflect the approximate blood lactate concentrations in mice (2.5 to 4.6 mM) (21, 22). At baseline, AAV8.Best1.MCT2-injected RPE samples had a significantly shorter lifetime compared to control, demonstrating that MCT2-expressing RPE cells have greater intracellular lactate levels when removed from the animal and not supplied with extracellular lactate (two-way ANOVA, *P* < 0.0001) (Fig. 4 *A* and *B*). When incubated with increasing concentrations of lactate, both control and AAV8.Best1.MCT2-injected samples had no detectable change at 0.1 mM compared to baseline [*P* = 0.7257 (control), *P* = 0.2901 (MCT2)]. However, both showed shorter lifetimes at 1 and 5 mM lactate, suggesting lactate uptake into these cells when supplied with these concentrations (*P* < 0.0001 for all conditions). AAV8.Best1.MCT2-injected samples also had significantly shorter lifetimes at each lactate concentration compared to control (*P* < 0.0001 for all conditions), suggesting that, despite having greater lactate levels at baseline, RPE cells expressing MCT2 continued to import lactate from the media, and imported more lactate than control RPE tissue.

**Fig. 5.**
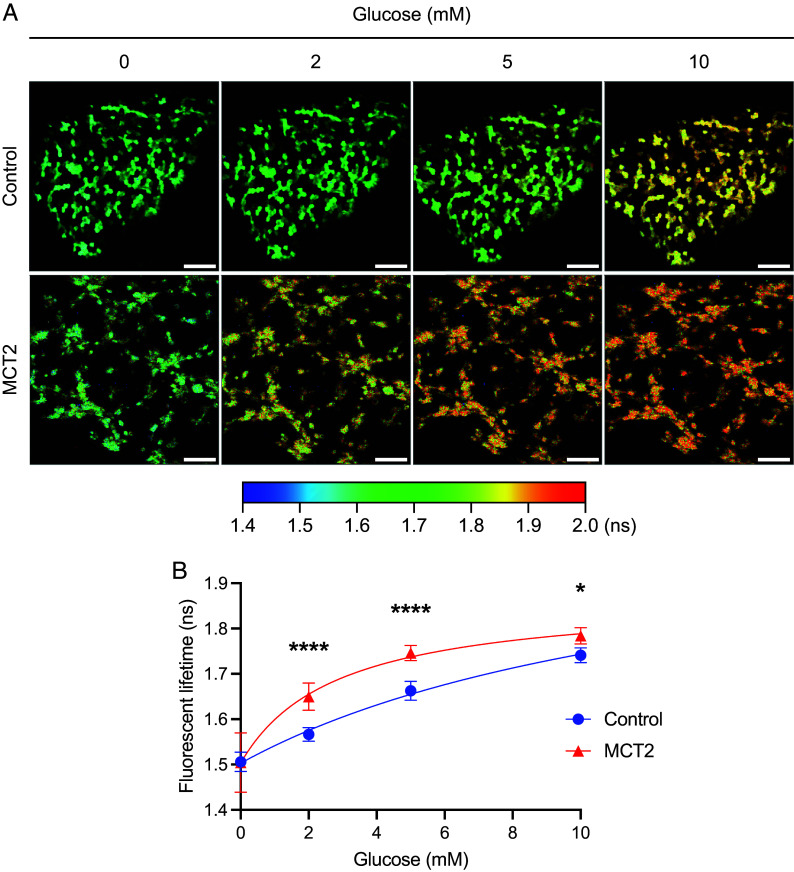
MCT2 overexpression leads to increased intracellular glucose in the RPE. Neonatal FVB mice were subretinally injected with AAV8.Best1.GlucoSnFR-TS alone (control) or coinjected with AAV8.Best1.MCT2 (MCT2). At P20-21, eyecups were harvested and RPE cells imaged en face using two-photon FLIM. (*A*). Representative lifetime images from control and MCT2 samples sequentially incubated with 0, 2, 5, and 10 mM glucose. Color scale represents increasing lifetime in ns, which corresponds to increased intracellular glucose concentrations. (Scale bar, 100 μm.) (*B*) Lifetime at 0, 2, 5, and 10 mM glucose (±SD; N_mice_ = 3; N_region_ = 9). Data fit with a dose–response curve. *P ≤ 0.05, ****P ≤ 0.0001 (two-way ANOVA with Šídák’s multiple comparison test).

**Fig. 6.**
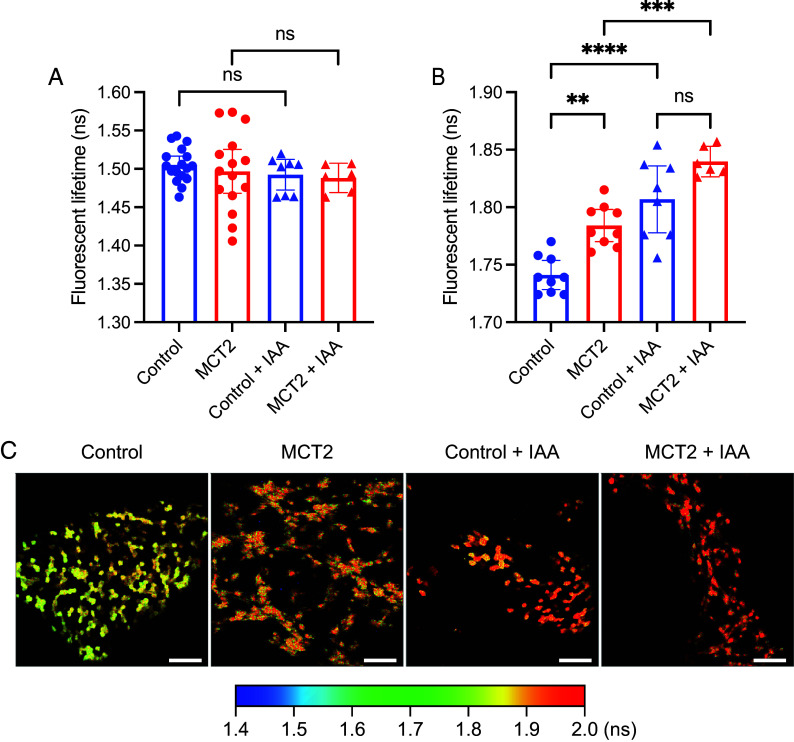
Inhibition of glycolysis increases intracellular glucose concentrations in the RPE. Neonatal FVB mice were subretinally injected with AAV8.Best1.GlucoSnFR-TS alone (control) or coinjected with AAV8.Best1.MCT2 (MCT2). At P20-21, eyecups were harvested and RPE cells imaged en face using two-photon FLIM. Following baseline lifetime recordings, the same RPE samples were treated with 0.5 mM IAA for 10 min. (*A*) Lifetime at baseline (0 mM glucose) with and without IAA treatment (±95% CI; control, n = 17; MCT2, n = 15; control + IAA, n = 8; MCT2 + IAA, n = 6). (*B*) Lifetime of RPE incubated with 10 mM glucose following treatment with or without IAA (±95% CI; control, n = 9; MCT2, n = 9; control + IAA, n = 8; MCT2 + IAA, n = 6). (*C*) Representative images from control and MCT2 samples incubated with 10 mM glucose and treated with and without IAA. (Scale bar, 100 μm.) **P ≤ 0.01, ***P ≤ 0.001, ****P ≤ 0.0001 (one-way ANOVA with Tukey’s multiple comparison test).

Next, we aimed to assess glucose levels as a readout for glucose uptake, glycogenolysis, and glycolysis. Neonatal FVB mice were subretinally injected with AAV8.Best1.GlucoSnFR-TS with and without AAV8.Best1.MCT2. Three weeks later, the RPE tissues were processed and incubated with 0, 2, 5, and 10 mM glucose in KRB buffer, which reflects the approximate blood glucose levels of FVB mice (9.1 mM) (40). At baseline, with 0 mM glucose in the media, there was no significant difference between the lifetime in control and AAV8.Best1.MCT2-injected samples, indicating that intracellular glucose levels were comparable (two-way ANOVA, *P* = 0.9998) (Fig. 5 *A* and *B*). This suggests that glycogenolysis, which would occur without added glucose, did not significantly raise the level of intracellular glucose when AAV8.Best1.MCT2 was delivered. However, upon incubation in increasing concentrations of glucose, the lifetime was significantly longer in the AAV8.Best1.MCT2-injected samples, showing greater intracellular concentrations of glucose [*P* < 0.0001 (2 and 5 mM), *P* = 0.0138 (10 mM)] (Fig. 5 *A* and *B*). As differences in intracellular glucose concentration could be due to differences in uptake, potentially due to changes in glucose transporter expression level, the level of glucose transporter 1 (GLUT1), the primary transporter for glucose in the RPE, was examined using immunohistochemistry. Stained retinal sections from FVB mice showed no detectable difference in GLUT1 expression when MCT2 was overexpressed (*SI Appendix*, Fig. S5). Another possible mechanism for higher intracellular glucose could be differences in the rate of glycolysis. To determine whether glycolysis was occurring in these cultures and whether a difference in its rate would lead to differences in intracellular glucose levels, we examined the effects of a blocker of glycolysis. The RPE samples were preincubated for 10 min with 0.5 mM of the GAPDH inhibitor, iodoacetic acid (IAA). At 0 mM glucose, there was no statistically significant difference in either the control or AAV8.Best1.MCT2-injected samples following IAA incubation, suggesting that, without added glucose, there was little glycolysis being performed (one-way ANOVA, *P* = 0.4642) (Fig. 6*A*). Following incubation with 10 mM glucose, there was a statistically significant increase in the fluorescent lifetime in both control (one-way ANOVA, *P* < 0.0001) and AAV8.Best1.MCT2-injected samples (*P* = 0.0003) (Fig. 6 *B* and *C*). This suggests that the ex vivo tissue culture conditions used in this study allow the RPE to import and metabolize glucose, allowing for the hypothesis that an increased intracellular concentration of glucose can be due to decreased glycolysis.

## Discussion

The current model for metabolic dysregulation in RP suggests that retinal lactate levels are substantially diminished due to the death of rods. This in turn causes the RPE to sequester glucose for its own metabolic needs and ultimately results in the starvation and degeneration of cones (9, 12). In this study, we used an AAV vector to express the high-affinity lactate transporter, MCT2, only in the RPE. The aim was to promote lactate uptake from the adjacent choroidal vasculature, which might then encourage the RPE to release more glucose for uptake by cones. Subretinal injections of an AAV8.Best1.MCT2 vector in rat and mouse models of RP promoted cone survival across several disease-causing mutations, as well as cone function. However, despite MCT2-expression increasing retention of cone function, this effect diminished with time, something which has been noted with other cone-rescuing therapies (3, 7, 13). It is likely that other mechanisms also contribute to cone degeneration, including inflammation (2–4) and oxidative damage (5–8). To achieve sustained rescue, each of these may need to be addressed in a combinatorial manner. Nevertheless, these data support the hypothesis that the metabolic status of the RPE impacts cone survival and reveals a gene-agnostic therapy for prolonging vision in RP. We cannot rule out, however, that MCT2 expression simply prevents atrophy of the RPE during retinal degeneration. As the RPE supports cone health and function, increased survival of RPE cells may nonspecifically increase cone survival. Regardless of the mechanism, MCT2 expression in the RPE provides for a retention of cone vision. Interestingly, a genome-wide association study found that a splice variant of MCT3 (*Slc16a8*) was correlated with age-related macular degeneration (41), while another found AAV-mediated overexpression of MCT2 in retinal ganglion cells in a glaucoma mouse model resulted in increased cell survival and metabolic improvements (42). These independent lines of evidence suggest that disrupted or inadequate monocarboxylate transport is associated with retinal diseases and suggest that there is therapeutic benefit for increased uptake.

A lack of appropriate techniques to assess the localization and levels of metabolites within the eye has made it challenging to understand the complexities of metabolism in healthy and diseased ocular tissues. The most commonly used techniques for measurements of metabolite levels have been mainly limited to whole tissue analyses. In this study, we applied FLIM-based biosensors for analysis of two metabolites in the eye at single-cell resolution. Moreover, as these sensors are genetically encoded, we could measure metabolites in a cell-type-specific manner. Using the FLIM-based biosensor for lactate, LiLac, we detected significantly greater intracellular lactate levels in AAV8.Best1.MCT2-infected RPE cells. The finding that there was more intracellular lactate when the RPE was first removed from the animal and incubated in media with no added lactate, is consistent with the most parsimonious explanation that MCT2 promoted more lactate uptake in vivo. It also appears that AAV8.Best1.MCT2-infected RPE was capable of more lactate uptake ex vivo, as intracellular lactate was higher in AAV8.Best1.MCT2-infected RPE relative to control when lactate was added to the media. More lactate might also result from the breakdown of glycogen, with subsequent glycolysis, but this would not be predicted to give higher lactate uptake when lactate was added to the media.

With the use of GlucoSnFR-TS we did not detect a difference in intracellular glucose levels at baseline, i.e., with no glucose added to the media. However, incubation with supplementary glucose resulted in a greater amount of glucose within MCT2-expressing RPE cells, which could be attributed to processes including reduced glycolysis, increased uptake, and increased glycogenolysis. An increase in glycogenolysis would not be predicted to be dependent upon glucose added to the media, so it is unlikely to be a significant contributor to the higher intracellular glucose. Regarding higher uptake, there is no reason to predict that MCT2 expression would directly affect glucose uptake and we did not see a difference in the expression level of the glucose transporter, GLUT1. Nonetheless, we cannot rule out an indirect effect on uptake. We were able to test whether reduced glycolysis could result in higher intracellular glucose. Treatment with the glycolysis inhibitor IAA, in conjunction with 10 mM glucose, resulted in a significant accumulation of intracellular glucose in both control and AAV8.Best1.MCT2-injected samples. As discussed above, MCT2-expressing RPE cells were seen to have increased intracellular lactate, which has been shown to inhibit glycolysis (17). Together, these findings are consistent with the hypothesis that MCT2-expressing RPE cells have higher intracellular glucose due to reduced rates of glycolysis.

Since the first step of glycolysis results in the irreversible retention of glucose, these findings support the model that cone degeneration is in part due to the RPE shifting from oxidative phosphorylation to glycolysis and withholding glucose for this purpose. IAA treatment, however, had no detectable effect on lifetimes in control or AAV8.Best1.MCT2-injected samples at baseline, suggesting that neither sample was performing glycolysis at the point when GlucoSnFR-TS measurements were first taken on freshly explanted tissue. Since RPE samples could perform glycolysis when glucose was added to the media, this suggests that very little intracellular glucose was present when the tissue was first explanted and assayed. GlucoSnFR-TS is highly specific to glucose and will not bind to any glycolytic intermediates, therefore the lack of detectable glucose at baseline may suggest residual levels were rapidly metabolized following tissue harvest (43). For this reason, the use of ex vivo tissue has its limitations. Although we analyzed tissue as quickly as possible after harvesting, the process of taking the tissue out of the organism likely impacts metabolism and may lead to depletion of some metabolites. Live imaging of the eye with adaptive optics fluorescent lifetime imaging ophthalmoscopy (AOFLIO) in conjunction with these FLIM-based biosensors, could enable a more accurate assessment of retinal metabolism in vivo (44, 45). Nonetheless, this technique demonstrates a significant improvement in the ability to detect metabolites in the eye. Overall, these data support the hypothesis for increased lactate uptake and reduced glycolysis in RPE cells as a possible therapy to support cone survival in RP.

Our aim in delivering MCT2 to RPE cells was for it to be expressed on the basolateral membrane to promote lactate uptake from the adjacent choroidal vasculature. Although we were able to detect MCT2 on this membrane, we also detected it on the apical membrane. The lower affinity transporter MCT1, which is also expressed on the apical membrane, functions to take up lactate produced by photoreceptors (19). The implication of MCT2 expression on the apical membrane is unclear and it may encourage additional uptake of lactate from the subretinal space. Furthermore, in addition to lactate, members of the MCT family can import other monocarboxylates including pyruvate and ketone bodies (33). Ketone bodies are produced by the RPE following metabolism of fatty acids acquired from phagocytosis of photoreceptor outer segments (46, 47). These ketone bodies are exported across the basal and apical membrane of the cell and can be taken up by photoreceptors via MCT1 and oxidized to carbon dioxide (19, 46). As MCT2 can also transport ketone bodies, it is possible that this has an additional effect on the metabolism of RPE and cones. Furthermore, the RPE does not solely rely on lactate as its source of nutrients, with several studies suggesting other potential fuel sources, including fatty acids (46, 48), glutamine (49), proline (50), and succinate (51–53). Although the data presented in this study suggest a possible role for lactate in RPE metabolism, further investigation into the other metabolites during cone degeneration should be explored.

We previously published that RPE-expressing AAV vectors induce ocular toxicity in mice (38). Interestingly, although we detected toxicity in mouse models subretinally injected with AAV8.Best1.MCT2, we did not observe significant toxicity in rats. As the rat eye is significantly larger than the mouse, it is possible that mice received a larger number of viral genomes per cell and, due to the dose-dependent nature of AAV-induced toxicity, larger doses would result in greater levels of toxicity (38). Nonetheless, we saw changes in RPE morphology in mice at doses as low as 1 × 10^8^ genome copies (gc)/eye, while minimal changes were seen at a dose of 2 × 10^9^ gc/eye in rats. Since the rat eye is only approximately double the size of a mouse eye, this 20-fold difference in dose is unlikely to account for the observed differences (54). These changes may be explained by a mouse-specific sensitivity to the overexpression of transgenes within the RPE. Although the mechanism behind this RPE toxicity remains unclear, there are several immune mechanisms lacking in the mouse which are seen in both rats and humans, such as a lack of major histocompatibility complex (MHC) class II molecules on T cells (55). Furthermore, we cannot rule out that MCT2 itself could be contributing to these morphological changes. MCT1-4 all couple lactate transport with a proton (33, 56), therefore changes in the expression of these transporters may alter intracellular pH and induce toxicity. For instance, in one study the knockout of MCT3 in mice was associated with a reduced electroretinogram (ERG), and in another, the RPE-specific knockout of basigin, which functions as a chaperone for MCT1 and MCT3 in their transport to the cell surface, induced reductions in ERG responses, disruption to photoreceptors, and changes to RPE morphology (57, 58). It is therefore possible that overexpression of MCT2 may be responsible for the mouse-specific toxicity. A means of assessing its effect on intracellular pH could be achieved using the genetically encoded pH sensor, pHRed, to detect lifetime changes and measure relative pH differences within MCT2-expressing RPE cells (59). Together, this raises the question about the choice of animal model when assessing both the efficacy and safety of potential therapeutics.

Overall, we demonstrate that AAV vector-mediated expression of MCT2 in RPE cells of rat and mouse models of RP improves cone survival and function across a range of mutations. We also present the use of FLIM-based biosensors in the eye that help uncover the mechanism of MCT2-mediated cone survival. The data suggest that this transporter is promoting lactate uptake and reducing retention of glucose. The development of FLIM-based biosensors for use within the eye may help elucidate the mechanism of other treatments targeting metabolic imbalances in RP and ultimately inform the design of future therapeutics. Together this supports the model for changes to RPE metabolism having an impact on cone survival in RP.

## Materials and Methods

### Animals.

S334ter line-3 rats (strain #00643) were purchased from Rat Resource & Research Center, University of Missouri (27). Sprague-Dawley rats (strain #001) were purchased from Charles River Laboratories. FVB (strain #207) and CD1 mice (strain #022) were purchased from Charles River Laboratories. P23H mice (strain #017628) were purchased from The Jackson Laboratory. Animals were bred and maintained at Harvard Medical School on a 12-h alternating light and dark cycle. All experimental procedures were approved by the Institutional Animal Care and Use Committee at Harvard University. Both males and females were used in all experiments and randomly assigned to groups.

### AAV Design and Production.

The vector AAV8.Best1.MCT2 was generated by replacing the GFP sequence in the AAV.Best1.GFP plasmid, which had been created by a fellow lab member, Wenjun Xiong (26, 38), with the mouse *Slc16a7* (MCT2) cDNA sequence. RNA was isolated from mouse retinae using the miRNeasy Mini Kit (QIAGEN) according to the manufacturer’s instructions. The RNA was reverse transcribed into cDNA using SuperScript® III First-Strand Synthesis Kit (Invitrogen) and purified using the QIAquick PCR Purification Kit (QIAGEN) according to the manufacturer’s instructions. The *Slc16a7* sequence was amplified using PCR and inserted into the AAV.Best1.GFP plasmid using Gibson cloning. AAV8.RedO.H2BGFP expressed a GFP fused to H2B and was under the control of a cone-specific RedO promoter (13, 28, 60, 61); this promoter was developed by Wang et al. (60); Li et al. (62) and obtained from the Botond Roska laboratory, Friedrich Miescher Institute for Biomedical Research, Basel, Switzerland (63). The vectors AAV8.Best1.LiLac and AAV8.Best1.GlucoSnFR-TS were generated from plasmids gifted by the Gary Yellen laboratory, Harvard Medical School (24, 25) and cloned into Best1 promoter plasmids (26). All AAV vectors were single stranded. AAV vectors were produced as previously described (7, 64). The vector plasmids were cotransfected with the Rep2/Cap8 packaging plasmid (pAAV2/8) and the adenoviral helper plasmid (pHGTI.adeno1).

### Subretinal Injections.

Subretinal injections were performed in neonatal animals at P0-2 as previously described (7, 65). In rats, 2 × 10^12^ gc/mL AAV8.Best1.MCT2 was coinjected with 0.01% red fluorescent beads (0.1 μm red FluoSpheres™, excitation/emission 580/605, Invitrogen) and 0.1% FastGreen (Millipore Sigma), in approximately a 1 μL volume per eye. For all rats, right eyes were injected with beads only, and left eyes were coinjected with AAV8.Best1.MCT2. In mice, AAV vectors were coinjected with 0.1% FastGreen, in approximately a 0.25 μL volume per eye. For all histology experiments performed in mice, 4 × 10^11^ gc/mL of AAV8.Best.MCT2 was delivered. For the P23H optomotor experiments 1 × 10^11^ gc/mL of AAV8.Best1.MCT2 was delivered. For FLIM experiments, 4 × 10^11^ gc/mL of AAV8.Best.MCT2 and 1 × 10^12^ gc/mL of either AAV8.Best1.LiLac or AAV8.Best1.GlucoSnFR-TS was delivered. In experiments using AAV8.RedO.H2BGFP 1 × 10^12^ gc/mL of vector was delivered.

### Histology.

Eyes to be used for cone counting were enucleated and dissected in PBS to remove the retina. For mouse retinal flatmounts, tissue was fixed in 4% paraformaldehyde (PFA) for 30 min at room temperature and then washed three times in PBS prior to mounting with Fluoromount-G mounting medium (Invitrogen). For rat retinal flatmounts, the tissue was used for HCR RNA-FISH using a protocol adapted from the manufacturer’s instructions with probes to *Arr3* (Molecular Instruments) (66, 67).

For retinal sections, eyes were dissected to remove the cornea, iris, and lens and leave the eyecup intact. Eyecups were cryoprotected by incubating in 7.5% sucrose until equilibrated before transferring to a solution of 50% OCT compound and 15% sucrose for at least 30 min. Eyecups were flash-frozen in this OCT/sucrose mixture and stored at −80 °C prior to sectioning with a Leica CM3050S cryostat (Leica Microsystems). Sections were hydrated by washing three times with PBS before incubating in LAB buffer (PolySciences Ltd) for 5 min at room temperature for a gentle antigen retrieval. Sections were washed once in PBS before incubating with blocking buffer [4% donkey serum, 0.3% triton x-100, 0.3% bovine serum albumin (BSA)] for 1 h at room temperature. Sections were incubated with a primary antibody against MCT2 (Proteintech; 20355-1-AP) at a 1/200 dilution or GLUT1 (Alpha Diagnostics; D1306) at a 1/300 dilution in blocking buffer overnight at 4 °C in a humidified chamber. The following day, sections were washed three times with PBS. The tissue was incubated with anti-rabbit 647 secondary antibody (Jackson ImmunoResearch) at a 1/750 dilution in blocking buffer for 2 h at room temperature in the dark. Sections were washed three times in PBS and stained with DAPI (Invitrogen) for 5 min before three subsequent washes. Sections were mounted in Fluoromount-G mounting medium.

For RPE flatmounts, after enucleation and removal of the cornea, iris, and lens, the retina was carefully removed to expose the apical surface of the RPE, attached to the choroid and sclera. The remaining RPE eyecup was fixed in 4% PFA for 2 h at room temperature, followed by three washes with PBS. The eyecups were incubated with 1/100 phalloidin Alexa Fluor 647 (Thermo Fisher Scientific) in blocking buffer (1% Triton-X and 1% BSA in PBS) overnight at 4 °C. The eyecups were washed three times in PBS and flattened with radial cuts and mounted onto a cover slip and slide.

### Image Acquisition and analysis.

All retinal flatmount images and the rat phalloidin-stained RPE were acquired on an Olympus VS200 Slide Scanner with a UPlan X Apo 10×/0.4 Air objective. FVB phalloidin stained RPE, GLUT1-stained FVB sections, and MCT2-stained S334ter sections were imaged on a Nikon Ti inverted microscope with a W1 Yokogawa Spinning disk with 50 μm pinhole disk and a Plan Apo λ 20×/0.75 DIC I objective. FVB and CD1 MCT2-stained sections were imaged on the Nikon Ti2 inverted microscope with a W1 Yokogawa Spinning disk with 50 μm pinhole disk and a Plan Apo λ 60×/1.4 Oil DIC objective.

GFP^+^ cone nuclei infected with AAV8.RedO.H2BGFP were imaged using the 488 nm laser and quantified using a modified version of an ImageJ macro as previously described (3, 4). In rats, we defined the transduced and untransduced area based on signal from the red fluorescent beads and the macro assigned four 250 μm^2^ polygons at the midpoint between the optic nerve and the outer retinal boundary in each of these regions (*SI Appendix*, Fig. S1). The cone count was the mean of these four regions. For mice, the same macro was used as previously described (3, 4), however, to limit the quantification to only the central retina, the region where the cones were counted was generated as ¼ of the distance from the optic nerve to the periphery rather than ½.

### Optomotor Assay.

Optomotor assays were performed using the OptoDrum (Striatech, Germany) automated system to determine the number of degrees per cycle. Mice were contralaterally injected with either AAV8.Best1.MCT2 and AAV8.RedO.H2BGFP, or AAV8.RedO.H2BGFP alone. All animals were habituated on the center platform for 5 min prior to the start of the first experiment. The black and white stripes were displayed at 99.7% contrast with the rotation speed of 12 degrees/s in both a clockwise or counterclockwise direction to assess the left and right eye, respectively. The OptoDrum software determined whether a mouse performed the corresponding head movement. The frequency of the stripes (cycles/degree) gradually increased until the mice failed to track three times.

### Two-Photon FLIM.

Eyes from mice aged P20-21 were enucleated in ice-cold HBSS and the cornea, iris, and lens were removed. One radial incision was made in the remaining eyecup and the retina was carefully removed to expose the RPE. An additional seven radial incisions were made, and the eyecup was transferred to KRB buffer (98.5 mM NaCl, 4.9 mM KCl, 2.6 mM CaCl_2_, 1.2 mM MgSO_4_, 1.2 mM KH_2_PO_4_, 26 mM NaHCO_3_, and 20 mM HEPES) before immediately proceeding with imaging (68, 69). The eyecup was transferred to a coverslip with the apical membrane of the RPE facing up. Residual buffer was wicked away using a Kimwipe to enable the eyecup to be gently flattened and the tissue was kept flat by using a slice anchor (Warner Instruments). The coverslip was transferred to the custom-made perfusion chamber prefilled with KRB buffer at room temperature. Lactate or glucose was delivered in KRB buffer through the perfusion system and samples were incubated for 5 min before data acquisition. Following IAA delivery, samples were incubated for 10 min.

Lifetime imaging was performed with a Leica upright DM6 Stellaris 8 Dive Falcon and a Plan Apo 25×/0.9 immersion objective. For LiLac, the light source was a two-photon tunable Insight X3 dual beam laser tuned to 850 nm at 8% laser intensity with a gain of 10 and emission light was bandpass filtered (465 to 550 nm). For GlucoSnFR-TS, the laser was tuned to 790 nm at 5% laser intensity with a gain of 10. For LiLac, 12 line accumulations were acquired for control samples and 15 for AAV8.Best.MCT2-injected samples. For GlucoSnFR-TS, 10 line accumulations were acquired for control and 10 to 15 for AAV8.Best.MCT2-injected samples. Phasor analysis was performed using the LAS X software (Leica) on data acquired from binning multiple Z stacks. The wavelet filter was applied to the phasor and the lifetime was measured at the center of the population (*SI Appendix*, Fig. S6).

A dose–response curve was fitted with a Hill equation (Eq. **1**) where data were plotted as lifetime (LT) against concentration of lactate or glucose (conc) (24, 25). LT_min_ and LT_max_ correspond to the lower and upper plateau, respectively, K_0.5_ is the concentration at the halfway point between LT_min_ and LT_max_, and n_H_ is the Hill coefficient, which was set to −1.0 for LiLac and 1.0 for GlucoSnFR-TS.

[1] $$LT={\mathrm{LT}}_{\min}+\frac{{\mathrm{LT}}_{\max}-{\mathrm{LT}}_{\min}}{1+{\left(\frac{K_{0.5}}{\mathrm{conc}}\right)}^{n_{H}}}.$$

### Statistics.

All statistical analysis was performed using GraphPad Prism Version 10. Datasets were tested for normality using a Shapiro–Wilk test. Data that were normally distributed were analyzed using a parametric test (*t* test or ANOVA), while skewed data were analyzed using a nonparametric test (Mann–Whitney test). Multiple comparisons were corrected for using Šídák’s or Tukey’s multiple comparison test for two- or one-way ANOVA, respectively. All statistical analyses were performed using an alpha of 0.05.

## Supplementary Material

Appendix 01 (PDF)

## Data Availability

All study data are included in the article and/or *SI Appendix*.
