# Supplementary material for: RPE-specific MCT2 expression promotes cone survival in models of retinitis pigmentosa
Source: Proc Natl Acad Sci U S A. 2025 Apr 3;122(14):e2421978122. doi: 10.1073/pnas.2421978122 (PMC12002273; doi:10.1073/pnas.2421978122)
Supplement: Supplementary file 1 — Appendix 01 (PDF) [file pnas.2421978122.sapp.pdf]

Supporting Information for

RPE-specific MCT2 expression promotes cone survival in models of retinitis pigmentosa

Laurel C. Chandler[a,b,c], Apolonia Gardner[a,b,c,d], Constance L. Cepko[a,b,c*]

[a]Departments of Genetics, Blavatnik Institute, Harvard Medical School, Boston, MA 02115.
[b]Departments of Genetics, Blavatnik Institute, Harvard Medical School, Boston, MA 02115.
[c]HHMI, Chevy Chase, MD 20815. [d]Virology Program, Harvard Medical School, Boston, MA 02115.
*Constance L. Cepko.

Email: cepko@genetics.med.harvard.edu

This PDF file includes:

Figures S1 to S6

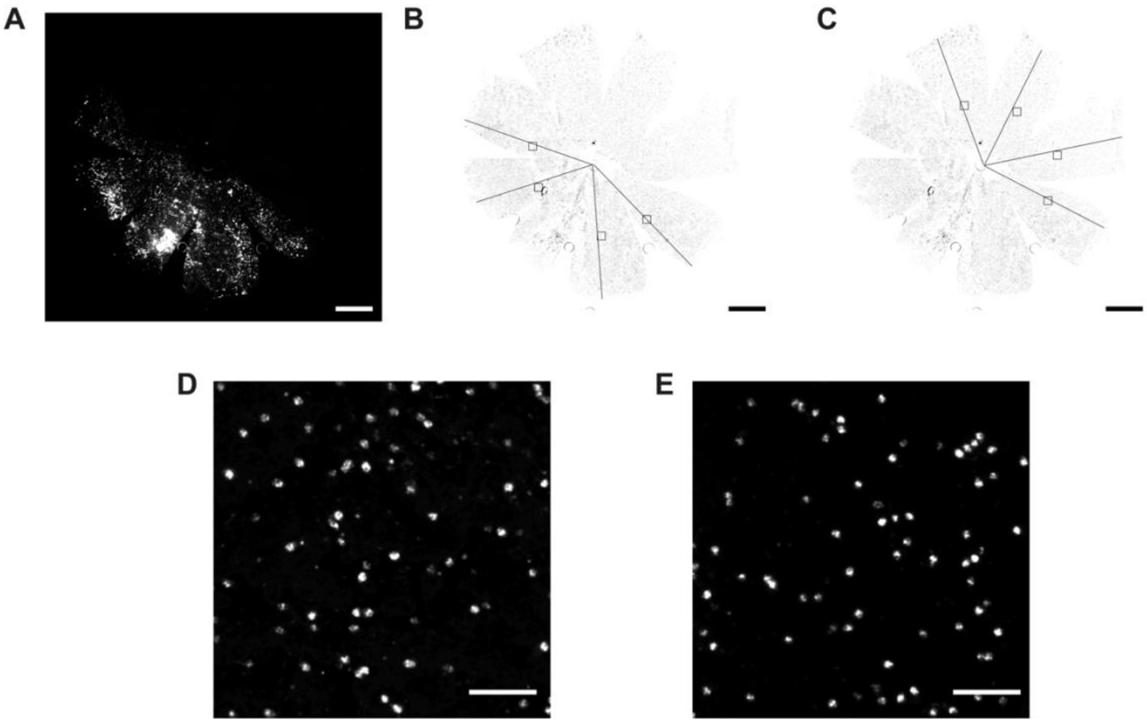

Figure S1. Method for cone counting in rat retinal samples. Representative retinae from a P180 S334ter rat subretinally injected with red fluorescent beads. Cones were stained using in situ hybridization for retinal cone arrestin-3 (*Arr3*). (A) Representative red fluorescent bead expression showing transduced (bead positive) and untransduced (bead negative) regions. (B) An ImageJ macro submitted *Arr3* stained retinal flatmount images to automatic processing and thresholding. Four lines were manually drawn from the periphery to the optic nerve head of the retina in the (B) transduced region and (C) untransduced region (A-C scale bar: 1 mm). 250 μm^2 boxes were automatically generated and placed along the midpoint of each line. *Arr3* stained cones in a single 250 μm^2 box in the (D) transduced and (E) untransduced region (scale bar: 50 μm^2). The mean number of cones in four 250 μm^2 boxes was calculated and used for statistical analyses.

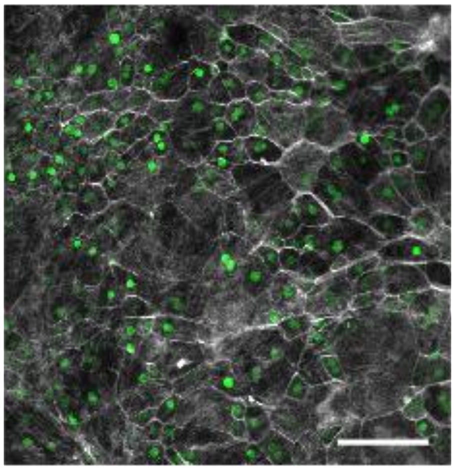

Figure S2. Expression of H2BGFP in the RPE. FVB mice were subretinally injected with AAV8.Best1.MCT2 (RPE-specific promoter) and AAV8.RedO.H2BGFP (cone-specific promoter). Representative image of phalloidin-stained RPE showing H2GFP expression in RPE nuclei demonstrating recombination or concatenation of the AAV8.Best1.MCT2 and AAV8.RedO.H2BGFP vectors (scale bar: 100 μm).

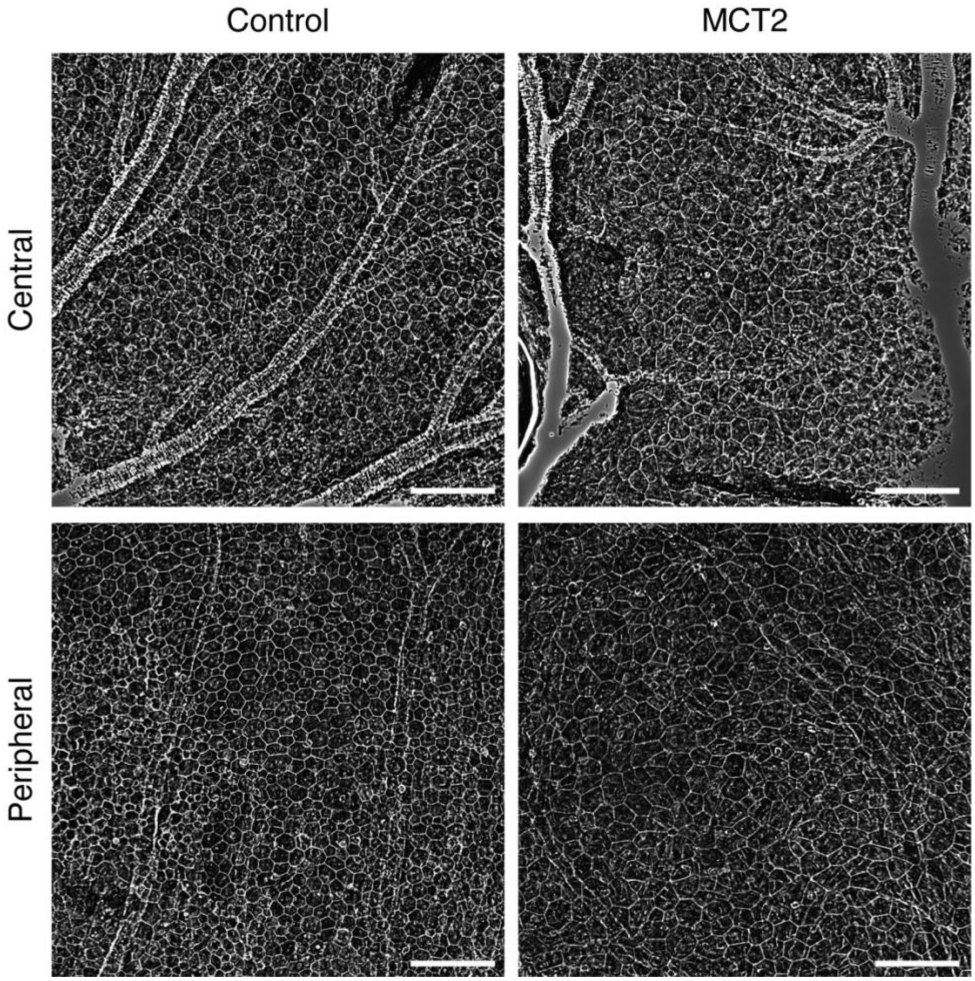

Figure S3. Minimal disruption to wildtype rat RPE following subretinal injection of AAV8.Best1.MCT2. Neonatal Sprague Dawley rats were subretinally injected with red fluorescent beads alone (control) or co-injected with AAV8.Best1.MCT2 (MCT2) (n=5). Representative images of P31 phalloidin-stained RPE at the central and mid-peripheral region showing minimal changes to the RPE hexagonal structure and no other signs of toxicity (scale bar: 100 μm).

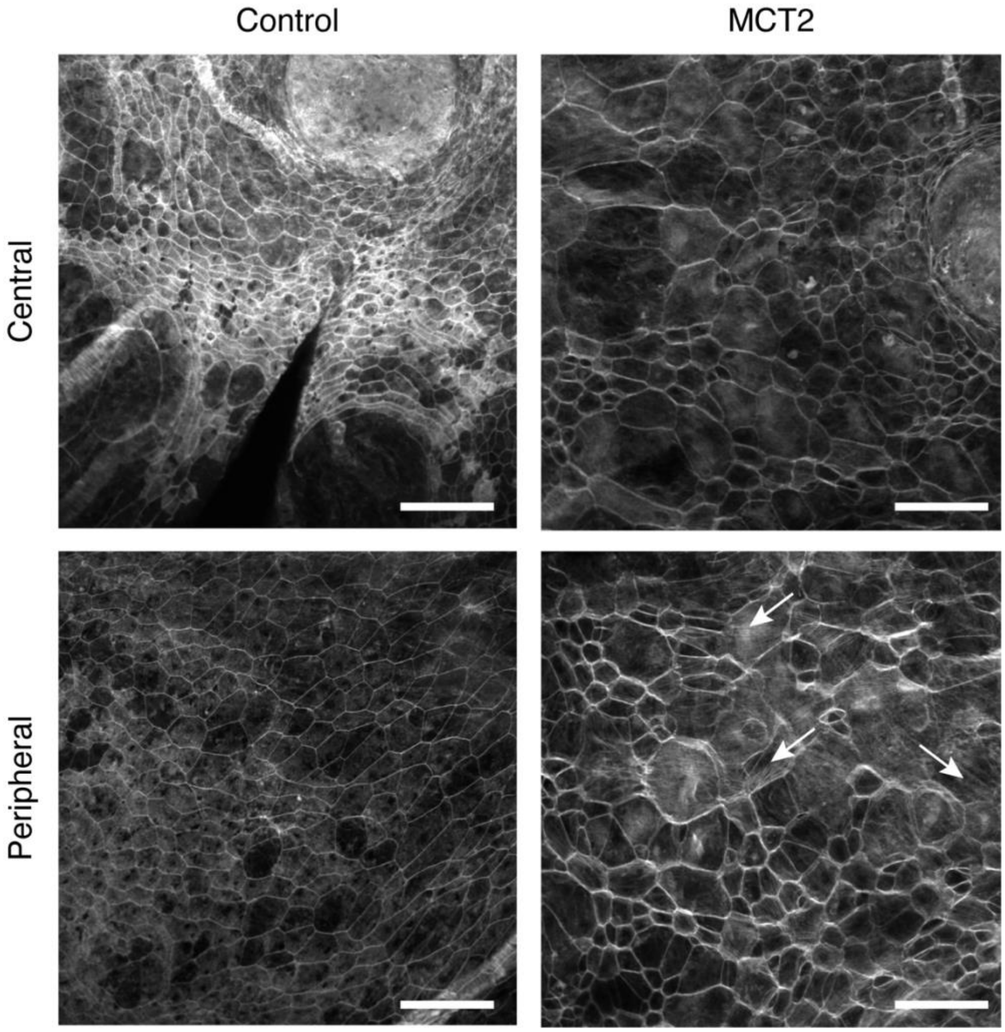

Figure S4. Signs of toxicity in mouse FVB RPE following subretinal injection of AAV8.Best1.MCT2. Neonatal FVB mice were subretinally injected with PBS (control) (n=5) or AAV8.Best1.MCT2 (MCT2) (n=7). Representative images of P40 phalloidin stained RPE at the central and mid-peripheral regions demonstrating increased disruption of RPE morphology and the upregulation of stress fibers (scale bar: 100 μm). Stress fibers are indicated with the white arrows.

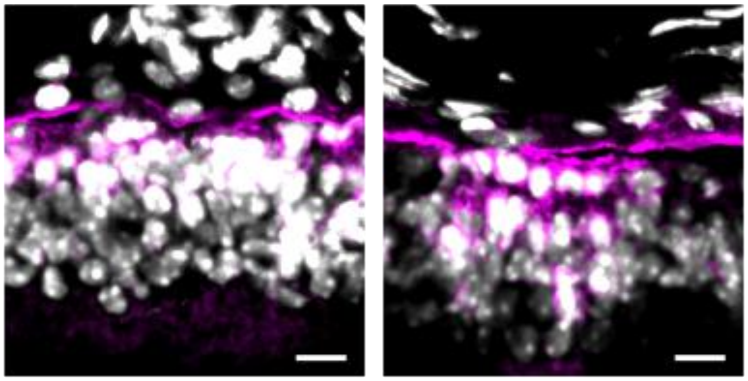

Figure S5. GLUT1 expression in FVB mice following AAV8.Best1.MCT2-injection. Representative section from a P42 FVB mouse subretinally injected with (MCT2) or without (control) AAV8.Best1.MCT2. All sections stained with GLUT1 antibody (magenta) and DAPI (white) (scale bar: 10 μm).

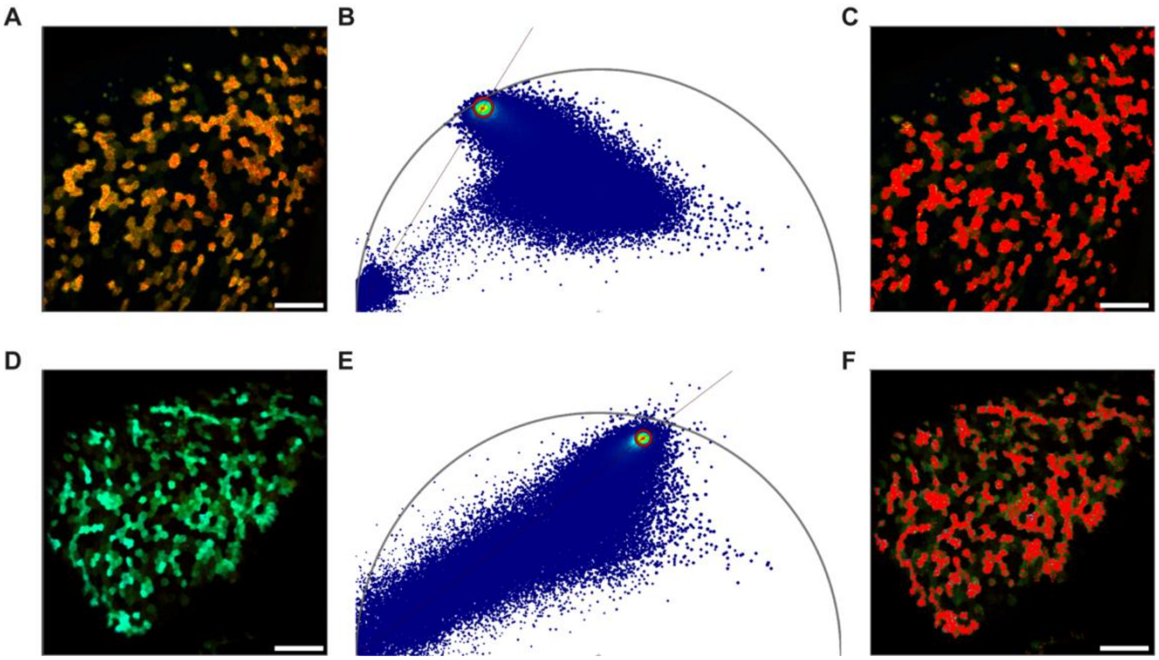

Figure S6. FLIM phasor analysis. RPE tissue from FVB mice subretinally injected with (A-C) AAV8.Best1.LiLac or (D-F) AAV8.Best1.GlucoSnFR-TS. (A&D) Representative lifetime image at baseline (scale bar: 100 μm). (B&E) The corresponding phasor plot demonstrating a 2D graphical view of lifetime distribution with each point on the plot corresponding to a pixel in the image. A single molecular species corresponding to (B) lactate or (E) glucose is selected within the red circle to calculate the lifetime of that sample. (C&F) Overlay in red demonstrating the subpopulation selected within the phasor plot.
